# A New Approach of Extraction of α-Amylase/trypsin Inhibitors from Wheat (*Triticum aestivum* L.), Based on Optimization Using Plackett–Burman and Box–Behnken Designs

**DOI:** 10.3390/molecules24193589

**Published:** 2019-10-05

**Authors:** Sorel Tchewonpi Sagu, Gerd Huschek, Josephine Bönick, Thomas Homann, Harshadrai M. Rawel

**Affiliations:** 1 University of Potsdam, Institute of Nutritional Science, Arthur-Scheunert-Allee 114-116, 14558 Nuthetal, Potsdam, Germany; sorelsagu@uni-potsdam.de (S.T.S.); homann@uni-potsdam.de (T.H.); 2 IGV-Institut für Getreideverarbeitung GmbH, Arthur-Scheunert-Allee 40/41, D-14558 Nuthetal OT Bergholz-Rehbrücke, Germany; gerd.huschek@igv-gmbh.de (G.H.); Josephine.Boenick@igv-gmbh.de (J.B.)

**Keywords:** wheat, α-amylase/trypsin inhibitors, extraction, Plackett–Burman design, Doehlert design, SDS-PAGE, MALDI-TOF/MS, LC-MS/MS

## Abstract

Wheat is one of the most consumed foods in the world and unfortunately causes allergic reactions which have important health effects. The α-amylase/trypsin inhibitors (ATIs) have been identified as potentially allergen components of wheat. Due to a lack of data on optimization of ATI extraction, a new wheat ATIs extraction approach combining solvent extraction and selective precipitation is proposed in this work. Two types of wheat cultivars (*Triticum aestivum* L.), *Julius* and *Ponticus* were used and parameters such as solvent type, extraction time, temperature, stirring speed, salt type, salt concentration, buffer pH and centrifugation speed were analyzed using the Plackett-Burman design. Salt concentration, extraction time and pH appeared to have significant effects on the recovery of ATIs (*p* < 0.01). In both wheat cultivars, *Julius* and *Ponticus*, ammonium sulfate substantially reduced protein concentration and inhibition of amylase activity (IAA) compared to sodium chloride. The optimal conditions with desirability levels of 0.94 and 0.91 according to the Doehlert design were: salt concentrations of 1.67 and 1.22 M, extraction times of 53 and 118 min, and pHs of 7.1 and 7.9 for *Julius* and *Ponticus*, respectively. The corresponding responses were: protein concentrations of 0.31 and 0.35 mg and IAAs of 91.6 and 83.3%. Electrophoresis and MALDI-TOF/MS analysis showed that the extracted ATIs masses were between 10 and 20 kDa. Based on the initial LC-MS/MS analysis, up to 10 individual ATIs were identified in the extracted proteins under the optimal conditions. The positive implication of the present study lies in the quick assessment of their content in different varieties especially while considering their allergenic potential.

## 1. Introduction

Wheat has become one of the most important foods in the world. Its growing consumption has led to an increase in allergies such as gluten intolerance, celiac disease and IgE-mediated allergies (including respiratory immune responses, especially for bakers) that affects about 0.4–2% of the general population, depending on the age and region [1,2,3]. A food allergy is described as an adverse health effect involving immunological mechanisms (allergic reactions) that can be induced in sensitized children and adults following dietary exposure to relevant allergens in foods [4]. These allergens are usually proteins, but sometimes chemicals such as haptens have also been reported [5]. Gluten has long been pointed out as being the main culprit [6,7,8,9]. Newer studies have explored other wheat compounds that may also cause such problems and one of these candidates are α-amylase/trypsin inhibitors (ATIs). ATIs are bifunctional biological substances that bind to α-amylase and trypsin making them inactive. They can inhibit bacterial amylases, insect amylase and mammalian amylase, and it has been further observed that some wheat ATIs strongly inhibits insect α-amylases, but not necessarily mammalian α-amylases [10]. Wheat ATIs generally contain five α-helices arranged in up and down manner and are rich in cysteine residues forming disulfide bonds. Wheat ATIs 0.19 and 0.28 were among the first to structurally be described; the inhibitor 0.19 being a dimer of molecular weight 24 kDa and the inhibitor 0.28 a monomer of molecular weight 12 kDa. [11]. Wheat ATIs were first studied in part because they played an important role in plant resistance to insects and microbial parasites [12] and also increasingly more recently because of their allergic effects [13,14,15].

Based on their differential solubility, wheat proteins can be classified into albumin and globulin, soluble in water or salt solutions, as well as gliadins (soluble in e.g., aqueous alcoholic solutions) and glutenins (in dilute acids/bases) remaining insoluble in water/salt solutions [16]. ATIs belong to the group of low-molecular weight salt-soluble proteins with masses ranging between 10 to 20 kD. As of September 2019, there were a total of 13 reviewed and two unreviewed wheat ATIs registered in the Uniprot online database (https://www.uniprot.org/uniprot, as assessed on 09.09.2019). They are characterized by their solubility in mixtures of chloroform and methanol (CM) and for this reason were called CM proteins [17]. As much as CM mixtures, salts solutions are also used for extraction of ATIs. Ammonium sulfate and sodium chloride at low concentration have been preferentially used [13,14,18]. Other buffer solutions were also employed. Geisslitz, et al. [19] used ammonium bicarbonate buffer to extract ATIs from 38 different products, including wheat, and fast-performance liquid chromatography was involved to complete the purification process. A number of studies have addressed the diversity of wheat ATIs by qualitative and quantitative characterization using HPLC fractionation and mass spectrometry tools. Feng et al. [12] analyzed α-amylase inhibitors from wheat flours by preparative, reversed-phase high performance liquid chromatography. Geisslitz et al. [19] used Targeted LC−MS/MS to quantify ATIs from different varieties of cereals, including wheat. Tundo, et al. [20] attempted to produce pure three ATIs considered major wheat allergens, named CM3, CM16 and 0.28, using isolated mRNA and cDNAs for heterologous expression.

It emerges from this review that there is a lack of data on optimization of ATI extraction. Empirical screening is generally used, which implies the use of numerous set of parameters until the appropriate conditions are achieved. This method is very expensive; it requires a great number of experiments and much time [21]. The establishment of a simple, cheaper and optimal method of ATIs extraction and purification remains a challenge. Design of experiments (DOE) is a systematic approach that uses mathematical tools to define the importance of specific processing variables and how to control them to optimize system performance while maximizing properties. DOE can generate the required information with a minimum of experimentation, using limited experiments, specific experimental conditions and mathematical analysis to predict the response at any point of the experimental domain [22]. In this work, a new approach to extract and purify ATIs from wheat in two steps is proposed. It combines the classical CM extraction with the selective precipitation using salt solutions. Different parameters are tested with the Plackett-Burman design and optimization is realized using Doehlert design.

## 2. Results and Discussion

### 2.1. Screening with Plackett Burman Design

Protein concentration and inhibition of amylase activity were evaluated with the Plackett Burman design and the results are presented in Table 1. Changes in protein concentration ranging from 0.16 ± 0.01 to 0.63 ± 0.04 mg/mL and inhibition of amylase activity (IAA) from 17.6 ± 0.9 up to 90.2 ± 0.7% were observed using *Julius*. About 0.16 ± 0.03 to 0.61 ± 0.04 mg/mL of the protein concentration, and 26.6 ± 1.6 to 90.3 ± 2.7% of IAA were registered with *Ponticus*.

#### 2.1.1. Evaluation of Parameters Effects on the Extraction of ATIs

Analysis of Plackett Burman design (PBD) experimental data was performed by calculating the coefficient of regression as well as the standard error and the statistical confidence level. The results are presented in Table S4 (Supplementary Data). It appears that the type of solvent mixture (X1), the composition of the solvent (X2) as well as the ratio of sample/solvent (X3) had no significant effect on the protein content and IAA as noted for *Julius* and *Ponticus*. These results show that dichloromethane can be used with the same performance as chloroform for carrying out ATIs extraction. Likewise, these results indicate that the ratios of chloroform/methanol or dichloromethane/methanol of 1:1 or 2:1 can be used without much difference in term of ATIs recovery. This corroborates the results showing that, although chloroform is considered to be a model hydrophobic polar solvent, dichloromethane can be used as a replacement for chloroform for the treatment of a large number of plant samples [23,24]. The use of mixtures of chloroform/methanol is a popular strategy used to extract highly hydrophobic proteins [25]. It is demonstrated that the C/M-soluble fractions from cereals esp. from wheat are enriched in proteins containing a high number of non-polar amino acids (49–59% not counting cysteine and tryptophan) [26].

The stirring speed and extraction temperature were not overall significant, with partly effects on few responses (Table S4). The same results were observed for urea, except result of IAA with *Ponticus* sample which was significantly different. Urea is generally used to denature protein complexes [27,28] and to reduce hydrophobic forces by providing an adequate environment for non-polar groups [29]. The ATIs being non-globular proteins, interactions of urea with hydrogen bonds or hydrophobic forces do not directly affect their stability in solution.

The negative sign of the coefficients of salt type and salt concentration indicates that increasing the values of these parameters had reduced the recovery of ATIs in solution. The clear understanding of salt effects on biological systems and in particular on interactions and phase behavior of proteins, what is meant by salting-out and salting-in, was always of great interest. It is well know that at low salt concentration, protein solubility generally increases (“salting-in”) whereas “salting-out” is an important experimental means of isolating, breaking down and crystallizing proteins occurring at high salt concentrations [30]. Thus, the behavior of proteins in solution will be directly related to the concentration of salt present. As can be seen, increasing of salt concentration helped to significantly reduce the protein content while maintaining the inhibitory activity, showing a selective precipitation of proteins (Table S4). Moller et al. [21] suggested that this is largely due to electrostatic screening effects which decrease the effective repulsive electrostatic interaction of the charged proteins and thus selectively reduce their solubility in water. Analysis reveals that at the same concentration, ammonium sulfate significantly reduces more protein concentration compared to sodium chloride. Minimum values of protein concentration of 0.16 ± 0.01 and 0.18 ± 0.01 mg/mL were obtained with ammonium sulfate at the concentration of 1 M using *Julius* (Table 1). It is well established that at equal concentrations, the ionic strength of ammonium sulfate is greater than that of sodium chloride, which justifies the observed results. Similar results were observed with pH. Table S4 show that pH had a significant influence on all experimental responses with *p*-value < 0.05. The structural integrity of proteins and their stability in solution depend on several factors and pH is one of the most important one. Either high or low pHs are widely used to precipitate proteins [31,32,33].

#### 2.1.2. Selection of Parameters for Optimization

Eleven parameters were screened by twelve trial runs using *Julius* and *Ponticus* as samples. Protein concentration and inhibition of amylase activity were used to evaluate the extraction process.

**Figure 1:**
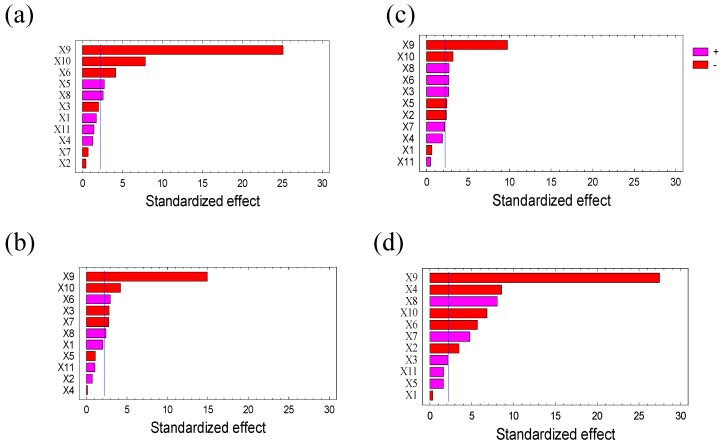
Standardized Pareto graph showing effect of the type of solvent, X1; the composition of solvents mixture, X2; the ratio of sample/solvent, X3; the concentration of Urea, X4; the extraction temperature, X5; the extraction time, X6; stirring speed, X7; pH, X8; type of salt, X9; concentration of salt, X10; and centrifugation speed, X11 on: (**a**,**b**) concentration of protein and (**c**,**d**) inhibition of amylase activity for Julius and Ponticus, respectively. The blue vertical line indicates the limit of the standardized effect whose values are significantly different from zero at the 95.0% confidence level. The pink color shows that the parameter has a positive effect and the red color a negative effect on the experimental response.

Based on the Pareto chart presented in Figure 1, parameters were classified according to their effects. It appeared that salt type, salt concentration, buffer pH, and extraction time were the most significant parameters (p < 0.01) (see also Table S4, Supplementary Data). The standardized effect showed a negative role of ammonium sulfate on protein concentration and IAA. For this reason, sodium chloride was therefore chosen to be used as precipitation salt. The salt concentration had a negative effect and needed to be optimized. The same effect was observed with extraction time and buffer pH. The efficiency of ATIs extraction and purification from wheat flour had to be increased by a lower salt concentration or a shorter extraction time. All these reasons led us to select the pH, the salt concentration and the extraction time as parameters to be optimized. Other parameters that did not significantly affect the extraction process were set based on the statistical analysis and their individual effects. A 2:1 ratio mixture of chloroform/methanol was selected as extraction solvent. The samples/solvent ratio of 125:1 (mass/volume), the stirring speed of 30 rpm, the extraction temperature of 4 °C and 10,000× g centrifugation speed of were chosen. Urea had no effect on ATIs extraction and was removed from the further optimization steps.

### 2.2. Optimization of Extraction Process

#### 2.2.1. Evaluation of the Effect of Parameters on Protein Concentration in IAA

Response surface methodology (RSM) with a Doehlert design (DD) was used to optimize the three most significant parameters: salt concentration, buffer pH and extraction time. Table 2 shows that the inhibition of trypsin activity was not substantial. Trends varied in total between 1.2 ± 0.1 and 6.6 ± 0.9% for *Julius* and *Ponticus*. Conversely, there was a significant variability in protein concentration and inhibition of amylase activity. For example, the protein contents varied from 0.22 ± 0.02 mg/L to 0.77 ± 0.05 mg/L (Table 2) and IAA from 37.1 ± 1.59 to 85.3 ± 2.5%. The corresponding distribution of the proteins fraction found and their molecular weights as estimated by SDS-PAGE analysis are given in Figure S2 (Supplementary Data).

**Figure 2:**
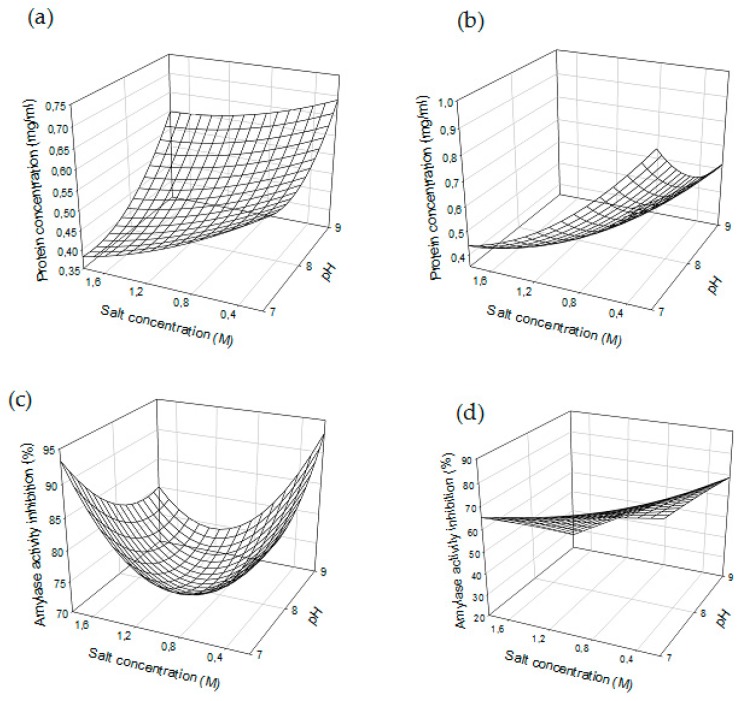
Response surfaces of (**a**,**b**) protein concentration and (**c**,**d**) amylase activity inhibition as function of salt concentration and pH with *Julius* and *Poncticus*, respectively. Extraction time is fixed at central point (105 min).

The prediction models giving the correlation between parameters and experimental responses were determined and their linear, quadratic and interaction coefficients are presented in Table 3. The coefficients of determinations for the protein concentration and IAA were 0.94, 0.91, 0.93 and 0.98 for *Julius* and *Ponticus*, respectively. The R^2^ statistic response indicates that the models as fitted are able to explain more than 90% of variability of the data. The accuracy factors, which are a qualitative term referring to measures of the relative average deviation between experimental and predicted responses were also estimated. They were 1.05 and 1.02, and 1.08 and 1.03 for *Julius* and *Ponticus*, respectively. The data shows that there was a significant correlation between different factors and experimental responses, which allowed validating the mathematical models. They were then used to represent the responses surface (Figure 2). A look at Table 3 raises the following observations: (1) Changes in salt concentration, extraction time and pH had a significant effect on protein concentrations, both using *Julius* and *Ponticus* (*p*-value < 0.01). As can be seen, the increase in sodium chloride concentration resulted in a reduction in protein concentration as much with *Julius* as with *Ponticus* (Figure 2a,b). (2) Factors that had a significant effect on IAA were only observed with *Ponticus*, while quadratic terms affected this response with *Julius*. The positive sign of those quadratic factors show the registered effects were positive. These suggest that although small variations in sodium chloride concentration and extraction time at pH values close to 7 had an impact on the extraction process, their variations at high levels tend not to more significantly affecting the extraction of ATIs. (3) Interactions between factors did not affect the system as much, an exception for the protein concentration with *Julius*. This demonstrates that each parameter worked independently and that there were no synergistic effects between them. It was not obvious to expect such a result, knowing that the selective precipitation of proteins is governed by the ionic strength and that salt concentration and pH are together involved in solutions ionic strength. Weighted effects of extraction time, sodium chloride concentration and pH expressed in percent were determined and consigned in Table S3 (Supplementary Data). The general observation is that while the sum of the linear effects accounted for about 30% of the completely observed effects with Julius, it exceeded 44% with *Ponticus*. Quadratic factors were almost 50% with *Julius* and only half (25%) for *Ponticus*. The interactions represented 21 and 31% in *Julius* and *Ponticus*, respectively.

#### 2.2.2. Numerical Optimization

Validated mathematical models were used to optimize the extraction process of ATIs from wheat flour. To this end, a numerical optimization was performed using the Statgraphics software (Statpoint Technologies, Inc., Warrenton, VA, USA). It was used to determine the combination of experimental parameters that simultaneously optimize responses with the best compromise. Since the results obtained with the inhibition of trypsin activity were not significant, only the results of protein concentration and IAA were processed. The goal was to minimize the protein concentration in order to precipitate the other proteins maintaining only ATIs in solution, and to maximize the inhibition of the amylase activity. Thus, solutions with maximal values of desirability of 0.94 and 0.91 were obtained for *Julius* and *Ponticus*, respectively. The optimal parameters values were: salt concentrations of 1.67 and 1.22 M, extraction times of 53 and 118 min, and pHs of 7.1 and 7.9 for *Julius* and *Ponticus*, respectively. The corresponding responses for both wheat varieties were: protein concentrations of 0.31 and 0.35 mg and IAAs of 91.6 and 83.3%. These optimal extraction conditions were tested in triplicate and the obtained p-values were all less than 0.05, indicating that the results were significantly different from zero at the 95.0% confidence level. The observed difference between optimal conditions with *Julius* and *Ponticus* could be due to their protein composition and therefore their ATIs composition (qualitatively and quantitatively). Dexter and Matsuo [34] showed that there is a difference in the proportion of non-gluten-related proteins (albumin and globulin) as well as a difference in the distribution of their solubility (Osborne scale) according to the wheat cultivars. In addition, growth conditions such as nitrogen, light, temperature and humidity greatly affect the differences in protein concentration between different cultivars [35].

**Figure 3:**
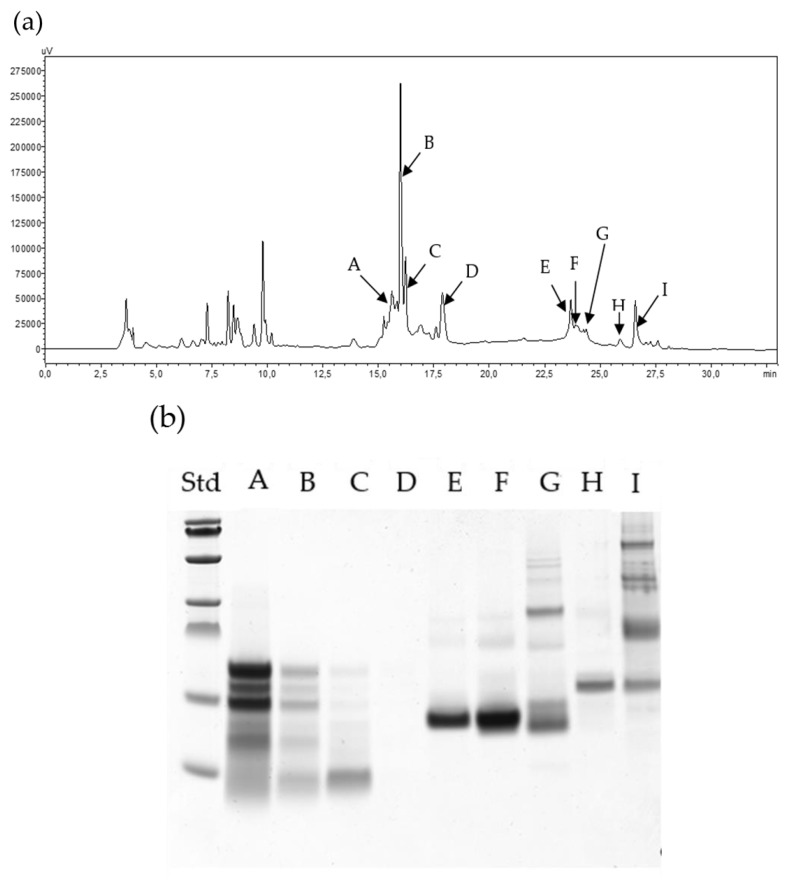

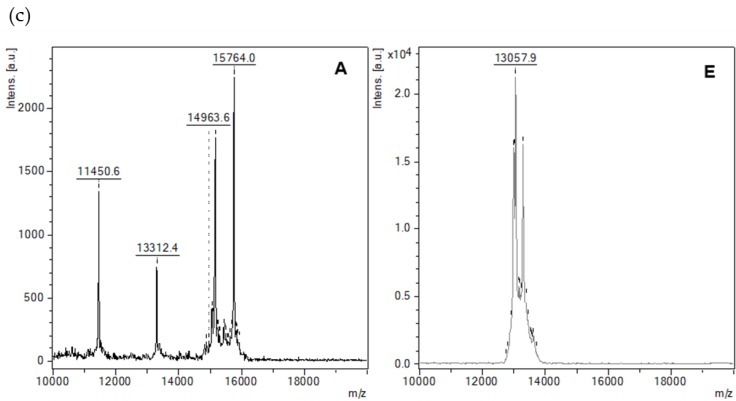
HPLC fractionation (**a**), SDS-PAGE (**b**) and MALDI TOF MS (**c**) of extracted ATIs from *Ponticus*.

**Figure 4:**
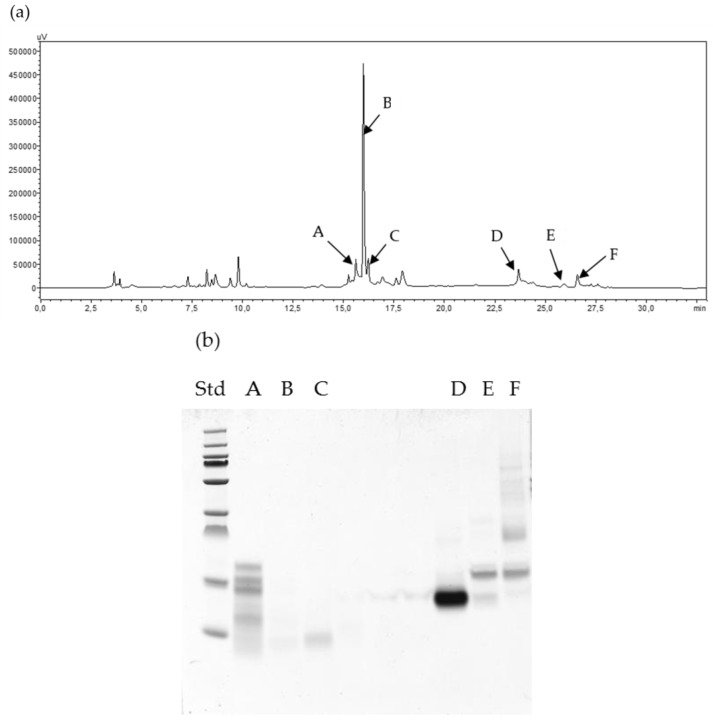

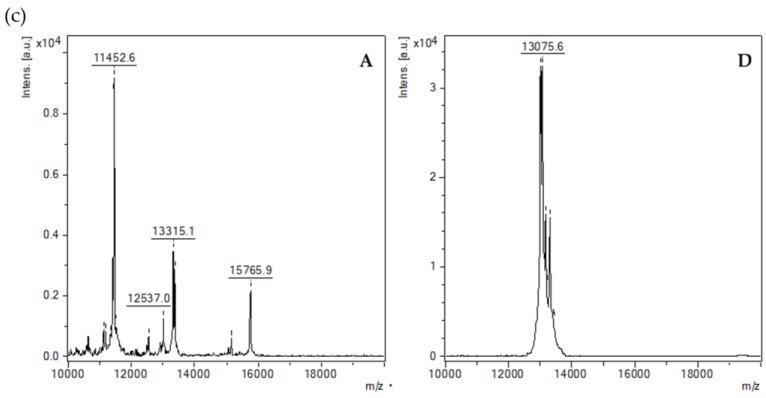
HPLC fractionation (**a**), SDS-PAGE (**b**) and MALDI TOF MS (**c**) of extracted ATIs from *Julius*.

The differences between the chromatograms obtained after ATIs fractionation of *Julius* and *Ponticus* are observed in Figure 3 and Figure 4. Electrophoresis showed that in both cases, the extracted ATIs had a mass ranging from 10 to 20 kDa. These masses correlate with those of UniProt online database, which currently includes 13 reviewed ATIs (https://www.uniprot.org/uniprot). In addition, MALDI analysis shown that a band on the gel electrophoresis can contains several ATIs of very close molecular masses. In the case of fractions D of *Julius* and E of *Ponticus*, single protein band was obtained, that allows a separation in several closely placed mass peaks with MALDI indicating different molecular species in the single band. The extracted proteins with optimized method were thereafter digested with α-chymotrypsin in order have a first impression of the ATIs present. The subsequent LC-MS analysis demonstrates that up to 10 ATIs can be identified (Table S5, Supplementary Data). The data shows a protein search summary and gives their accession numbers and numbers of the identified peptides for each protein. It also supplies the sequence coverage in each case. On basis of this primary data, further experiments are being carried out to enable quantification.

## 3. Materials and Methods

### 3.1. Materials

The two soft winter varieties of wheat, *Julius* and *Ponticus* (14.6% and 14.1% total proteins as per Kjedahl method), harvested in 2018 were received from IGV-Institut für Getreideverarbeitung GmbH, Nuthetal OT Bergholz-Rehbrücke, Germany. Chloroform (99.8%), dichloromethane (99.8%), methanol (100%), sodium chloride and ammonium sulfate (99%) were purchased from VWR Chemicals (Leuven, Belgium). Urea was obtained from Merck KGaA (Darmstadt, Germany). Folin & Ciocalteu’ phenol, potassium sodium tartrate tetrahydrate and maltose monohydrate (99%) were acquired from Sigma-Aldrich (Steinheim, Germany). Tris and petroleum ether bp 30–75 °C were obtained from Carl Roth GmbH (Karlsruhe, Germany). 3,5-Dinitrosalicylic acid, 97+% was obtained from Alfa Aesar GmbH (Karlsruhe, Germany). Iodoacetamide (IAA) (PubChem CID: 3727) as well chymotrypsin from bovine pancreas for enzymatic digestion were bought from Sigma Aldrich (St. Louis, MO, USA). Reagents and solutions were all prepared from chemicals by dilution with distilled water.

### 3.2. Extraction of ATIs

The diagram describing the different steps of ATIs extraction from wheat samples is presented in the Figure S1 (see Supplementary Data). Wheat flour (100 mg) was mixed with petroleum ether (0.4 mL) for 10 min at 30 rpm. After centrifugation at 10,000× *g*, 4 °C for 5 min (centrifuge 5415R, Eppendorf AG, Hamburg, Germany), the supernatant was removed and the precipitate was air dried at room temperature for about 20 min. The extraction solvent was added and the whole was stirred according to the conditions derived from the experimental design. Mixture of chloroform/methanol and dichloromethane/methanol with different ratios were applied. After centrifugation at 10,000× *g*, 4 °C for 5 min, the supernatant was fully collected and dried using a centrifugal evaporator (RC10-22, Jouan S.A., Saint-Herblain, France). Samples were redissolved in 25 mM Tris buffer solution containing salt, and after vortex, sonication for 5 min and centrifugation (10,000× *g*, 4 °C for 5 min), the supernatant containing purified ATIs (fraction with low molecular weight proteins) was carefully collected and transferred to the new micro-tubes. The precipitate containing high molecular weight protein fraction was re-dissolved in 1% sodium dodecyl sulfate solution for analysis. Ammonium sulfate and sodium chloride were tested and different parameters were evaluated to determine the optimal conditions of the extraction process.

### 3.3. Experimental Designs

DoE combining Plackett-Burman design (PBD) and Doehlert design (DD) were used to screen and optimize the extraction process of ATIs.

#### 3.3.1. Plackett–Burman Design to Screen Parameters

PBD is a two-level statistical design, which allows a rapid analysis of a large number of parameters in a minimum number of runs. It identifies with a high degree of precision the most critical parameters that significantly affect the process and those that have a negligible impact on the results. It also allows the analysis at the same time of both qualitative quantitative parameters [36]. In this study, eleven parameters were analyzed with PBD: the type of extraction solvent (X1, chloroform/methanol or dichloromethane/methanol), the composition of solvents mixture in ratio (X2, 1:1 and 2:1; volume/volume), the ratio of sample/solvent (X3, 125:1–250:1 masse/volume), the concentration of Urea (X4, 0–50 mg/mL), the extraction temperature (X5, 4–25 °C) and the extraction time (X6, 60–180 min), stirring speed (X7, 40–80 rpm), the buffer pH (X8, pH 8–pH 9), the type of salt (X9, ammonium sulfate and sodium chloride), the concentration of salt (X10, 0.5–1 M) and centrifugation speed (X11, 10,000–12,000 g). Preliminary studies as well as data from the literature were used to select parameters and their levels. Twelve experiments were generated (Table 1) and each experiment was made in duplicate randomly, the analysis carried out and data reported as mean ± standard deviation.

#### 3.3.2. Doehlert Design

The Doehlert design (DD) was chosen for optimization. It has a uniform distribution of experimental points and is a rotatable design with constant variance at equidistant from the central point. The experimental domain can be extended by adding new independent variables or moving the design to a new experimental domain [37]. DD requires a smaller number of experiments while obtaining better precision of coefficient estimates and the number of levels studied for each variable changes: five levels for the first parameter, three for the last and seven for the second in the case of a matrix with three variables [38]. Three parameters to be optimized in the DD were selected from the PBD results and their ranges of variation are described in Table S1 (supplementary data). The DD allows the description of a region around an optimal response with n^2 + n + 1 points for n parameters. With three parameters, seventeen experiments with four replications at the central point were generated and corresponding real values were calculated according to the coded values given by the DD (Table 2).

### 3.4. High-Performance Liquid Chromatography

The extracted ATIs were fractionated with a Shimadzu HPLC system (Shimadzu Europa GmbH, Duisburg, Germany). Solutions of 0.1% trifluoroacetic acid (TFA) and 70% acetonitrile (ACN) were used as eluent A and B, respectively. Elution was carried out at a flow rate of 1 mL/min and at different solvent gradients. 100 μL of ATIs extracted under optimal conditions were applied to a 300 × 4.6 mm PerfectSil 300 ODS C18 5 μm column (MZ Analysentechnik, Mainz, Germany). Solvent gradient during HPLC fractionation is presented in Table S2 (Supplementary data). The fractions were collected 5 times each, pooled, dried and then re-dissolved in 100 μL of 25 mM Tris buffer, pH 8. The re-dissolved fractions were tested for their inhibitory activity, SDS PAGE and MALDI-TOF-MS.

### 3.5. Analyses

Protein concentration, inhibition of amylase (IAA) and inhibition of trypsin (ITA) were used as experimental responses to evaluate the performance of the extraction process.

#### 3.5.1. Determination of Protein Concentration

Concentration of protein was determined by the method of Lowry, et al. [39] with some modifications. Bovine serum albumin (Fluka Chemie AG, Buchs, Switzerland) was used as standard.

#### 3.5.2. Inhibition of Amylase Activity

Inhibition of amylase activity was done by the DNS method [40] with modifications. The positive test was made by mixing of 10 µL of 1 mg/mL α-amylase—specific activity of 23 units/mg solid (Sigma Aldrich, Steinheim, Germany) with 190 µL of 0.02 M sodium phosphate buffer, pH 6.9 with 0.006 M sodium chloride. The blank was prepared with 200 µL of sodium phosphate buffer. After pre-incubation at 37 °C for 3–4 min to achieve temperature equilibration, 200 µL of 1% starch solution were added and the mixture incubated for 15 min at 37 °C. The reaction was stopped by adding 400 µL of 2,3 dinitrosalicylic acid reagent (DNS). The mixture was heated at 99 °C for 5 min to develop the color, cooled down at room temperature, the total volume filled to 1.5 mL with 700 µl of distilled water and the absorption was read at 540 nm with a spectrophotometer (Hanna instruments, Vöhringen, Germany). Inhibition assay was done in the same way by pre-incubating 10 µl of amylase solution with 70 µl of extracted ATIs and 120 µL of sodium phosphate buffer at 37 °C for 10 min. A negative test was prepared with 70 µL of ATIs and 130 µL of sodium phosphate buffer. Calibration was done using maltose solutions ranging from 0.25 to 5 µmol/mL. Residual activity was calculated and inhibition of amylase activity (IAA) expressed in percent was evaluated as described in Equation (1):

*IAA* (%) = [(*AA*_C_-*AA*_T_)/*AA*_C_] × 100 (1)

with *AA*_C_ the amylase activity of the control (without ATIs) and *AA*_T_ the tested amylase activity in presence of purified ATIs at the same conditions.

#### 3.5.3. Trypsin Activity and Determination of it Inhibition

Modified azocasein protease assay as described by Sagu, et al. [41] with some slight modifications was used to evaluate the trypsin activity inhibition. 75 µL of extracted ATIs were mixed with 30 µL of 0.5% sodium bicarbonate buffer pH 8.3 and 20 µL of 10 mg/mL trypsin solution (Sigma Aldrich). For the control, 20 µL of trypsin were mixed with 105 µL of buffer. 125 µL of 2.5% azocasein solution (Sigma Aldrich) were added and the mixture was incubated at 37 °C for 30 min. 100µL of the mixture were mixed with 400 µL of 5% trichloroacetic acid solution, incubation at room temperature for 5 min and centrifuged for 5 min at 10,000 g. 400 µL of the above solution were mixed with 1200 µL of 500 mM NaOH solution and the absorbance was taken at 440 nm. The trypsin activity was expressed as the amount of activity that gives a change of one unit of absorbance at 440 nm/min, and the inhibition of trypsin activity (ITA) calculated in percent according to the Equation (2):

*ITA* (%) = [(*TA*_C_-*TA*_T_)/*TA*_C_] × 100 (2)

where, *TA*_C_ is the trypsin activity of control and *TA*_T_ the tested trypsin activity in presence of ATIs.

#### 3.5.4. Electrophoresis

Electrophoresis [42] was done under reducing and denaturing conditions using Invitrogen NuPAGE 12% Bis-Tris precast protein gels with 12 wells (Thermo Fisher Scientific, Carlsbad, CA, USA). 15 µL of extracted ATIs were mixed with 15 µL NuPAGE™ LDS Sample Buffer (1×) pH 8.4 containing glycerol, 2-mercaptoethanol, SDS and Coomassie blue G250. The mixture was heated at 100 °C for 5 min to denature and reduce protein disulfide bonds. After cooling to room temperature, 15 µL of samples and 5 µL of pre-stained broad range standard containing nine protein ladders (10, 15, 25, 35, 55, 70, 100, 130 and 250 kDa; Thermo Fisher Scientific) were loaded in the gel and the separation was carried out under constant current (30 mA/Gel) for about 120 min using NuPAGE MES SDS Running Buffer (1×). Gels were stained overnight with Coomassie brilliant blue R-250 solution and then distained with 10% acetic acid. Gels were scanned with Bio-5000 Professional VIS Gel Scanner (SERVA Electrophoresis GmbH, Heidelberg, Germany) and analyzed with Image Lab Software (Bio-Rad Laboratories Ltd., Hemel Hempstead, UK).

#### 3.5.5. MALDI-TOF-MS Analysis

ATIs were analyzed for their molecular weight with a MALDI-TOF MS instrument (Autoflex Speed LRF, Bruker Daltonik GmbH, Bremen, Germany) [43]. 2 µL of samples were mixed with 2 µL of 2% TFA and 2 µL of 2,5-Dihydroxy actetophenone (Alfa Aesar, Heysham, UK) and aliquots of 2 µL were applied to the target plate (MTP target frame III, Daltonik). Protein Standard I (Bruker Daltonics) containing a mixture of four proteins was used for internal calibration and the measurements were performed in linear mode (LM).

#### 3.5.6. Protein Digestion and Untargeted Triple TOF/MS-Analysis

The protein digestion is based on our former reports [44,45]. Briefly, the extracted ATIs were incubated with iodoacetamide for 20 min at 50 °C in the dark to alkylate cleaved disulfide bonds. The overnight-digestion at 25 °C was initiated by adding chymotrypsin (dissolved in 1 mM HCl) and stopped by adding 40% formic acid. To purify and isolate the peptides, a solid phase extraction using C18 cartridges (Chromabond, Machery Nagel, Düren, Germany) was performed. The columns were activated using a mixture of 50% ACN and 0.1% formic acid and conditioned with triple distilled water before application of the digested extracts. After a washing step with acidified water (0.1% formic acid) the peptides were eluted using 50% ACN/0.1% formic acid. The eluate was evaporated under nitrogen to achieve complete dryness and re-suspended using 50% ACN/0.1% formic acid and 0.1% formic acid. Untargeted peptide analysis was performed with a high resolution Triple TOF/MS system, consisting of an Eksigent micro UHPLC system (Applied Biosystems/MDS Sciex, Concord, ON, Canada), an PAL HTS-xt system auto sampler CTC Analytics (CTC Analytics, Zwingen, Switzerland) and a TripleTOF © 5600 MS/MS (AB Sciex Germany GmbH, Darmstadt, Germany). To separate the peptides a C18 analytical micro column (Halo fused C18 column, 2.7 µm, 90A, 0.5 mm × 50 mm, Eksigent) was used. Digested samples were diluted 1:100 with 0.1% formic acid. The HPLC gradient program starts with 95% solvent A (0.1% formic acid in water) and increases after 36 min to 30% solvent B (0.1% formic acid in acetonitrile), after 41 min to 60% and from 42 min to 46 min to 90% at a flow rate of 0.15 µL/min and an oven temperature of 35 °C. From 47 min until the end of the program (51 min) the organic solvent stays at 5% for re-equilibration. Ionization was performed in positive ESI mode. Ion spray voltage was set to 5500 V and curtain gas (CUR) at 25 units. Full scan spectra were measured between 400 and 1200 Da. Product ion spectra were measured in IDA between 200 and 1800 Da for ions with charge state 2–5 and which exceed 150 cps. To identify the ATIs, the acquired data was compared with 15 databank entries for Amylase Trypsin Inhibitors (organism: Triticumaestivum) in UniProtKB (http://www.uniprot.org) with the ProteinPilot software using the Paragon Algorithm. The resulting data were submitted to a False Discovery Rate-Analysis and resulted in 9 matches (FDR < 1%).

### 3.6. Statistical Analysis

Quadratic polynomial equations with interaction between parameters were established to describe the effect of variables on experimental responses as predictive models:

*Y* = *b*_0_ + *b*_1 × 1_ + *b*_2 × 2_ + *b*_3 × 3_ + *b*_11 × 1_^2^+ *b*_22 × 2_^2^+ *b*_33 × 3_^2^ + *b*_12 × 1_*X*_2_ + *b*_13 × 1_*X*_3_ + *b*_23 × 2_*X*_3_ (3)

with *X*_1,_ *X*_2_ and *X*_3_ the parameters, *b*_0_ the constant, *b*_1_, *b*_2_ and *b*_3_ the first-order coefficients, *b*_11_, *b*_22_ and *b*_33_ the second-order coefficients and *b*_12_, *b*_13_, *b*_23_ the interaction coefficients. The least squares method was used to determine coefficients. The analysis of the variance (ANOVA) was performed and the *p*-values were calculated to evaluate the significance of the results. Effects with *p*-values less than 0.05 indicate that they are significantly different from zero at the 95.0% confidence level. Pareto chart was used to classify the parameters according to their effects. It highlights the most significant variables and facilitates the selection of those to optimize.

In addition to the coefficient of determination (*R*^2^), the accuracy factor (*A*_F_) was determined in order to validate the proposed models. This analysis criterion evaluates the difference between the estimated and experimental values of the variables and was calculated as follows:

*A*_F_ = 10^(Σlog|*Y*i,est/*Y*i,exp|/N)^ (4)

where, *Y*_i,est_ is the estimated response, *Y*_i,exp_ the experimental response and N the number of experiments. Generation of the experimental Designs and statistical analysis were performed with Statgraphics software (Statpoint Technologies, Inc., Warrenton, VA, USA) and the response surfaces were plotted with SigmaPlot software (Systat Software Inc., San Jose, CA, USA).

## 4. Conclusions

The salt type, salt concentration, buffer pH and extraction time were found to be the most important factors affecting the solubility of ATIs. Proteins were precipitated more selectively with sodium chloride than with ammonium sulfate. Chloroform and dichloromethane both produced similar results in both cultivars; therefore, dichloromethane would be a more preferable alternative while considering the negative health related effects of chloroform. Analysis of the variance showed that there was no significant interaction between the salt concentration, pH and extraction time during the optimization process. The wheat cultivars *Julius* and *Ponticus* presented only slightly different optimal conditions. The summarized optimal extraction parameters, as well as the corresponding inhibitory activities and protein concentrations from the two tested wheat varieties, are presented in Table S6 (Supplementary Data). These conditions therefore may also be used for other wheat cultivars. Further work is going on to this respect. The selective extraction of the α-amylase/trypsin inhibitors is not well studied and therefore in the present manuscript a simple and optimal method was developed considering different aspects and factors which may influence their extractability. The positive implication of the present study lies in the quick assessment of their content in different varieties especially while scrutinizing and selecting wheat cultivars with low potential allergenic effects. HPLC allowed further fractionation and molecular weights of purified ATIs were determined by electrophoresis and MALDI-TOF-MS. Up to 10 ATIs have been identified per LC-MS out of a total of 15 reviewed and unreviewed ATIs currently listed in the Uniprot online database. In a further study, the characterization of the individual ATIs present in different cultivars with focus on their compositional quantification using targeted and untargeted proteomics tools is being envisaged.

## Figures and Tables

**Table 1:** Plackett-Burman design presenting the eleven factors and the two corresponding experimental responses for the *Julius* and *Ponticus* cultivars.

Nb	Parameters											Experimental Responses			
												*Julius*		*Ponticus*	
	X_1_	X_2_	X_3_	X_4_	X_5_	X_6_	X_7_	X_8_	X_9_	X_10_	X_11_	Protein conc. (mg/mL)	IAA (%)	Protein conc. (mg/mL)	IAA (%)
1	D/M	2:1	125:1	50	4	60	30	9	(NH4)_2_SO_4_	1	10000	0.22 ± 0.03	29.3 ± 1.6	0.27 ± 0.03	26.6 ± 1.6
2	D/M	1:1	250:1	0	4	60	80	9	(NH4)_2_SO_4_	0.5	12000	0.31 ± 0.01	63.3 ± 2.2	0.27 ± 0.01	65.0 ± 3.0
3	C/M	2:1	250:1	50	4	180	80	8	(NH4)_2_SO_4_	0.5	10000	0.20 ± 0.04	61.9 ± 5.3	0.24 ± 0.08	29.0 ± 1.4
4	D/M	2:1	250:1	0	25	180	30	9	NaCl	0.5	10000	0.63 ± 0.04	87.8 ± 3.2	0.61 ± 0.04	90.3 ± 2.7
5	D/M	1:1	250:1	50	4	180	30	8	NaCl	1	12000	0.49 ± 0.01	90.2 ± 0.7	0.52 ± 0.01	62.0 ± 2.6
6	C/M	1:1	250:1	50	25	60	80	9	NaCl	1	10000	0.56 ± 0.05	89.8 ± 2.4	0.40 ± 0.03	87.0 ± 3.5
7	C/M	2:1	250:1	0	25	60	30	8	(NH4)_2_SO_4_	1	12000	0.18 ± 0.01	17.6 ± 0.9	0.16 ± 0.03	35.7 ± 1.0
8	C/M	1:1	125:1	50	25	180	30	9	(NH4)_2_SO_4_	0.5	12000	0.32 ± 0.01	56.0 ± 1.7	0.35 ± 0.03	39.5 ± 0.9
9	C/M	2:1	125:1	0	4	180	80	9	NaCl	1	12000	0.50 ± 0.02	85.1 ± 5.2	0.53 ± 0.05	83.4 ± 1.7
10	D/M	1:1	125:1	0	25	180	80	8	(NH4)_2_SO_4_	1	10000	0.16 ± 0.01	29.8 ± 4.1	0.21 ± 0.02	33.8 ± 1.9
11	C/M	1:1	125:1	0	4	60	30	8	NaCl	0.5	10000	0.62 ± 0.07	78.7 ± 2.5	0.55 ± 0.01	87.3 ± 2.7
12	D/M	2:1	125:1	50	25	60	80	8	(NH4)_2_SO_4_	1	12000	0.23 ± 0.02	20.4 ± 0.8	0.19 ± 0.01	26.6 ± 1.4

C: chloroform; D: dichloromethane; M: methanol; IAA: inhibition of amylase activity. With X1—the type of extraction solvent, X2—the composition of solvents mixture in ratio, X3—the ratio of sample/solvent, X4—the concentration of Urea, X5—the extraction temperature, X6—time of extraction, X7—stirring speed, X8—the pH of Tris buffer, X9—type of salt, X10—concentration of salt and X11—centrifugation speed.

**Table 2:** Doehlert design and experimental responses for optimization of ATIs extraction.

Nb							Experimental Responses					
	Coded Values			Real Values			*Julius*			*Ponticus*		
				*X*_10_	*X*_6_	*X*_8_	Protein Concentration	*IAA*	*ITA*	Protein Concentration	*IAA*	*ITA*
	*x*_10_	*x*_6_	*x*_8_	(M)	(min)	(pH)	(mg/mL)	(%)	(%)	(mg/mL)	(%)	(%)
1	0	0	0	1	105	8	0.50 ± 0.05	73.1 ± 0.5	1.6 ± 0.6	0.48 ± 0.09	60.4 ± 3.1	3.8 ± 0.1
2	1	0	0	1.8	105	8	0.41 ± 0.05	82.2 ± 4.7	2.0 ± 0.1	0.32 ± 0.02	44.5 ± 2.3	2.1 ± 0.1
3	−1	0	0	0.2	105	8	0.58 ± 0.02	83.7 ± 1.2	3.3 ± 0.2	0.37 ± 0.05	76.3 ± 1.0	3.5 ± 0.3
4	0.5	0.866	0	1.4	180	8	0.38 ± 0.03	81.0 ± 1.5	3.0 ± 0.5	0.65 ± 0.05	48.8 ± 0.8	5.8 ± 1.0
5	−0.5	−0.866	0	0.6	30	8	0.49 ± 0.01	83.7 ± 3.6	1.8 ± 0.0	0.36 ± 0.13	49.1 ± 0.7	6.6 ± 0.9
6	0.5	−0.866	0	1.4	30	8	0.37 ± 0.02	84.2 ± 3.0	4.7 ± 0.9	0.77 ± 0.15	40.3 ± 1.1	7.1 ± 1.1
7	−0.5	0.866	0	0.6	180	8	0.53 ± 0.03	83.3 ± 0.9	2.2 ± 0.3	0.66 ± 0.16	60.9 ± 0.1	5.3 ± 0.8
8	0.5	0.289	0.816	1.4	130	9	0.47 ± 0.04	75.0 ± 1.0	2.3 ± 0.9	0.49 ± 0.02	37.1 ± 1.5	6.4 ± 0.5
9	−0.5	−0.289	−0.816	0.6	80	7	0.61 ± 0.03	80.9 ± 2.7	4.0 ± 0.2	0.79 ± 0.18	72.3 ± 1.7	5.7 ± 0.6
10	0.5	−0.289	−0.816	1.4	80	7	0.36 ± 0.03	85.3 ± 2.5	5.0 ± 0.3	0.34 ± 0.15	67.1 ± 1.1	3.5 ± 0.2
11	0	0.577	−0.816	1	155	7	0.52 ± 0.02	77.5 ± 0.7	4.3 ± 0.7	0.40 ± 0.09	69.4 ± 1.2	4.1 ± 0.2
12	−0.5	0.289	0.816	0.6	130	9	0.68 ± 0.02	82.5 ± 1.4	1.2 ± 0.1	0.58 ± 0.05	57.8 ± 2.1	3.6 ± 0.5
13	0	−0.577	0.816	1	55	9	0.56 ± 0.04	79.3 ± 0.4	3.0 ± 0.2	0.33 ± 0.06	42.3 ± 2.5	3.5 ± 0.8
14	0	0	0	1	105	8	0.43 ± 0.04	75.4 ± 2.1	1.8 ± 0.5	0.42 ± 0.02	59.7 ± 4.2	4.4 ± 0.4
15	0	0	0	1	105	8	0.51 ± 0.05	69.8 ± 4.3	2.2 ± 0.6	0.39 ± 0.01	62.2 ± 1.9	2.9 ± 0.3
16	0	0	0	1	105	8	0.45 ± 0.01	73.7 ± 0.7	1.5 ± 0.1	0.43 ± 0.09	61.9 ± 2.5	3.9 ± 0.5
17	0	0	0	1	105	8	0.47 ± 0.03	65.7 ± 1.5	1.3 ± 0.1	0.41 ± 0.08	58.9 ± 2.3	3.6 ± 0.2

With, *X*_10_ showing the concentration of sodium chloride, *X*_6_ the extraction time and *X*_8_ the pH. *IAA* is the inhibition of amylase activity and *ITA* is the inhibition of trypsin activity. Each experiment was conducted in triplicate. The average values reported along with standard deviation are provided.

**Table 3:** Factor coefficients estimated after analysis of Doehlert design and their corresponding R^2^ and *A*_F_.

Factors	Coeff.	*Julius*		*Ponticus*	
		Proteins conc. (mg/mL)	IAA (%)	Proteins conc. (mg/mL)	IAA (%)
***Constants***					
	b0	0.472	71.5	0.406	60.6
***Linear***					
*X*_10_	b_1_	−0.071 **	−1.0	−0.212 ***	−13.8 ***
*X*_6_	b_2_	0.086 **	−1.7	−0.115 *	5.1 **
*X*_8_	b_3_	0.102 ***	−1.4	−0.149 **	−14.6 ***
***Quadratic***					
*X*_10_* *X*_10_	b_11_	0.023	11.4**	0.089	−0.2
*X*_6_* *X*_6_	b_22_	−0.047	11.5**	0.156 *	−14.4
*X*_8_* *X*_8_	b_33_	0.098 **	7.1*	0.112	−0.8
***Interaction***					
*X*_10_* *X*_6_	b_12_	−0.017	−1.6	−0.208 *	−2.0
*X*_10_* *X*_8_	b_13_	0.031	−6.7	0.049	−8.8 *
*X*_6_* *X*_8_	b_23_	−0.039	3.6	0.356 **	−1.1
*R^2^*		0.94	0.91	0.93	0.98
*A_F_*		1.05	1.02	1.08	1.03

* Significant at *p* ≤ 0.05, ** significant at *p* ≤ 0.01, *** significant at *p* ≤ 0.001. *X*_6_ is extraction time, *X*_8_ the pH and *X*_10_ sodium chloride concentrations. *A_F_* is the accuracy factor.

## Supplementary Materials

The following are available online, Figure S1: Extraction process of ATIs using mixtures of chloroform/methanol (C/M) or dichloromethane/methanol (D/M) as extraction solvents, Figure S2: Table S1: Protein profiles of (a) *Julius* extract and (b) *Ponticus* extract for different experimental conditions of the Doehlert design determined by SDS-PAGE, Table S1: Experimental factors and their level in Doehlert design, Table S2: Solvent gradient during HPLC fractionation, Table S3: Weighted effects of extraction time sodium chloride concentration and pH on experimental responses expressed in percent, Table S4: Factor coefficients estimated after analysis of Doehlert design and their corresponding R^2^ and *A_f_*.

## Abbreviations

ACN	Acetonitrile
*A*_F_	Accuracy factor
AA_C_	Amylase activity of the control
AA_T_	Tested amylase activity
*b*_0_, *b*_1_, *b*_2_, …	Coefficients of models
ATIs	α-amylase/trypsin inhibitors
C/M	Chloroform/methanol mixture
D/M	Dichloromethane/methanol mixture
DD	Doehlert design
DNS	Dinitrosalicylic acid color reagent
DoE	Design of experiment
IAA	Inhibition of amylase activity
ITA	Inhibition of trypsin activity
LM	Linear mode
M	Molarity
MALDI-TOF	Matrix Assisted Laser Desorption Ionization-Time of Flight
MS	Mass Spectrometry
*N*	Number of parameters
*p*	Probability
PBD	Plackett - Burman design
SDS	Sodium dodecyl sulfate
*X*_1,_ *X*_2,_ *X*_3_	Parameters
TA_C_	Trypsin activity of control
TA_T_	Tested trypsin activity
TFA	Trifluoroacetic
*Y*	Experimental response
2,5-DHAP	2,5-Dihydroxy actetophenone
