# Supplementary material for: A New Approach of Extraction of α-Amylase/trypsin Inhibitors from Wheat (Triticum aestivum L.), Based on Optimization Using Plackett–Burman and Box–Behnken Designs
Source: Molecules. 2019 Oct 5;24(19):3589. doi: 10.3390/molecules24193589 (PMC6803881; doi:10.3390/molecules24193589)
Supplement: Supplementary file 1 [file molecules-24-03589-s001.pdf]

Supplementary Material

A new approach of extraction of α-amylase/trypsin inhibitors from wheat based on optimization using Plackett–Burman and Box–Behnken designs

Sorel Tchewonpi Sagu [1], Gerd Huschek [2], Josephine Bönick[1,2], Thomas Homann[1] and Harshadrai M. Rawel[1,]*

1. Institute of Nutritional Science, University of Potsdam, Arthur-Scheunert-Allee 114-116, 14558 Nuthetal, Potsdam, Germany; sorelsagu@uni-potsdam.de (S.S.) ; homann@uni-potsdam.de (T.H.)

2. IGV-Institut für Getreideverarbeitung GmbH, Arthur-Scheunert-Allee 40/41, D-14558, Nuthetal OT Bergholz-Rehbrücke, Germany; gerd.huschek@igv-gmbh.de (G.H.)

* Corresponding author.

Tel: +49-33200-88-5525/5578 (H.R.)

E-mail: rawel@uni-potsdam.de

Content

Figure S1. Extraction process of ATIs using mixtures of chloroform/methanol (C/M) or dichloromethane/methanol (D/M) as extraction solvents

Figure S2. Protein profiles of (a) *Julius* extract and (b) *Ponticus* extract for different experimental conditions of the Doehlert design determined by SDS-PAGE

Table S1. Experimental factors and their level in Doehlert design

Table S2. Solvent gradient during HPLC fractionation

Table S3. Weighted effects of extraction time sodium chloride concentration and pH on experimental responses expressed in percent

Table S4.Factor coefficients estimated after analysis of Doehlert design and their corresponding R^2 and A_f

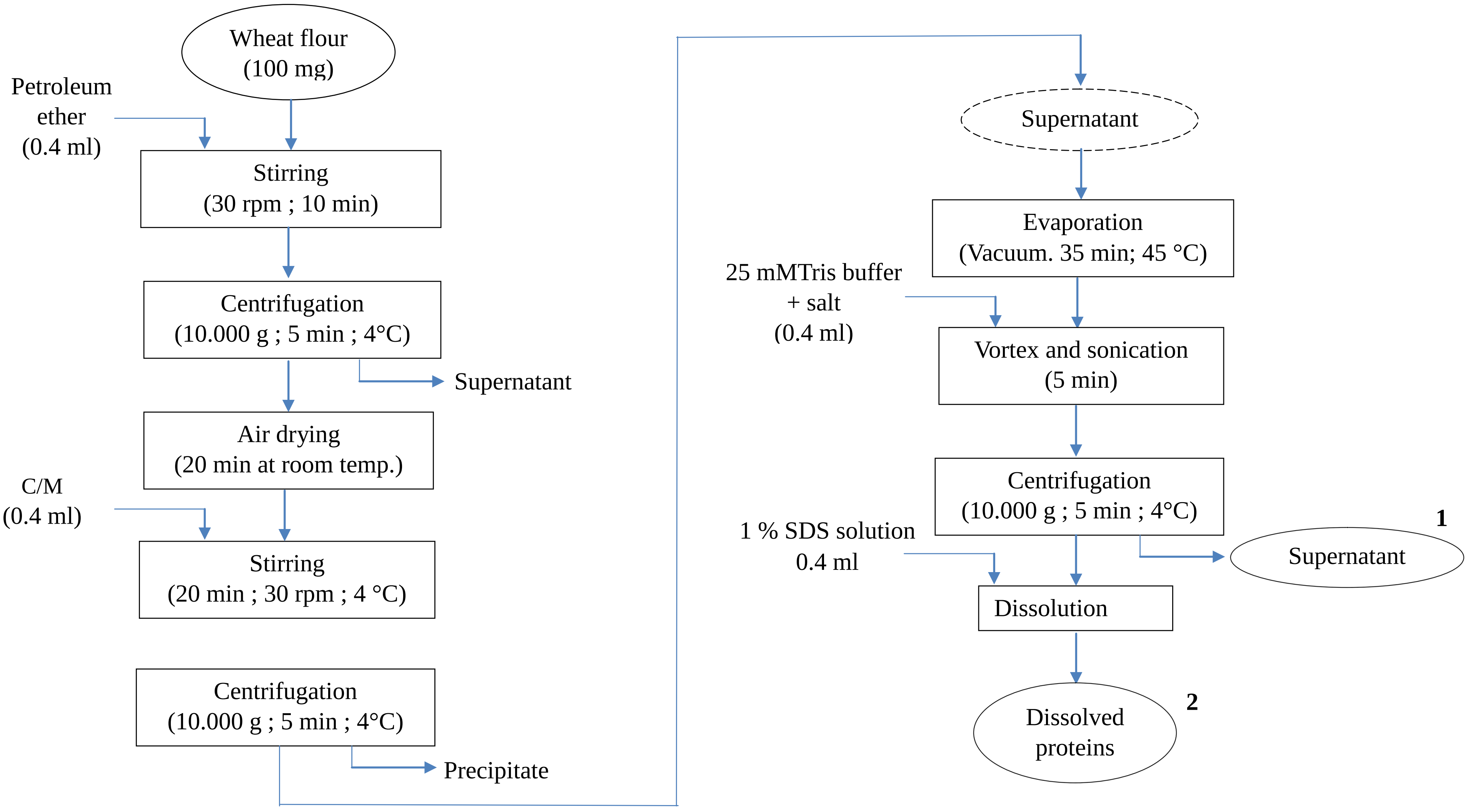

Figure S1. Process flow chartof wheat amylase/trypsin extraction using mixtures of chloroform/methanol (C/M) or dichloromethane/methanol (D/M) as extraction solvents. Sample 1 contains the ATIs and Sample 2 contains other proteins of higher molecular weights.

(a)

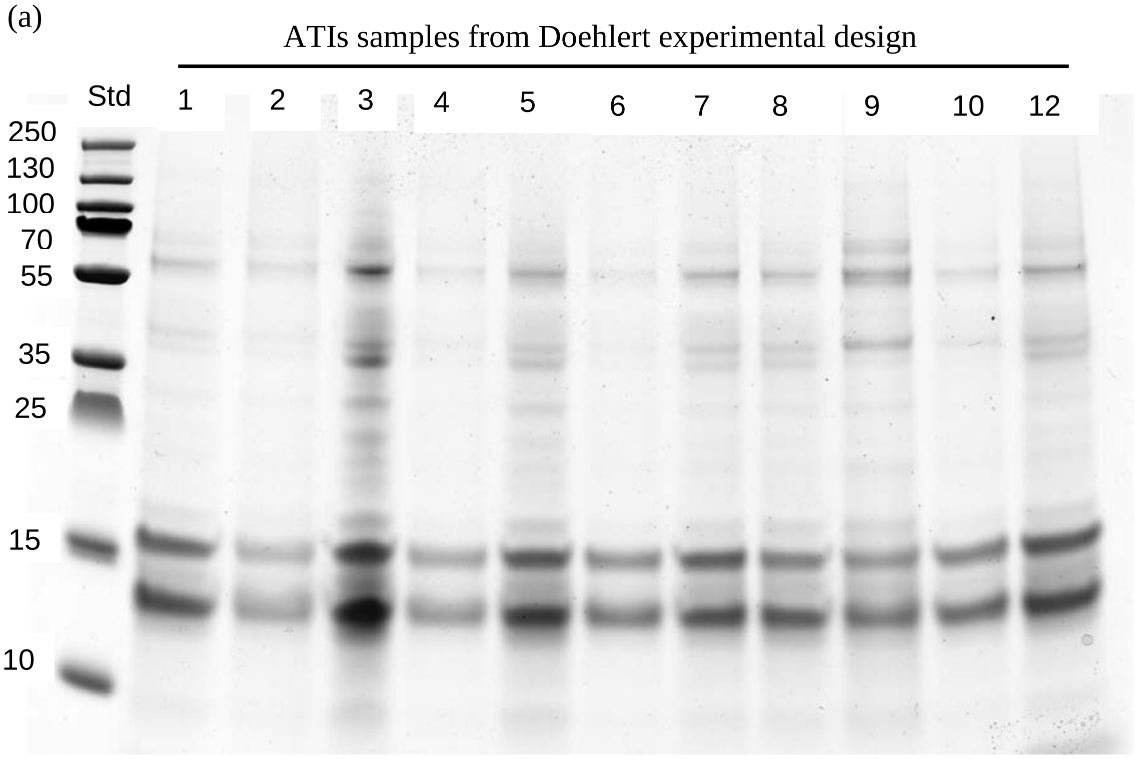

(b)

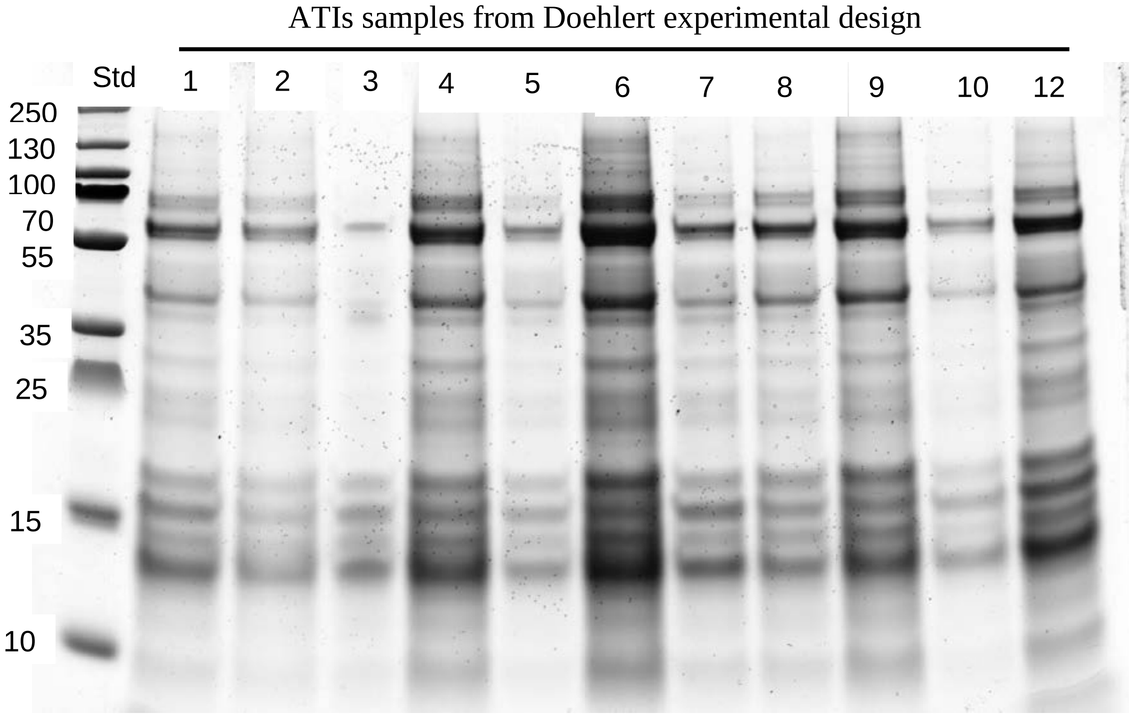

Figure S2. Protein profiles of selected (a) *Julius* extract and (b) *Ponticus* extract for different experimental conditions of the Doehlert design determined by SDS-PAGE

Table S1. Experimental factors and their level in Doehlert design

Factor	Factor significance	Low level	High level
X_6	Extraction time (min)	30	180
X_8	pH (/)	7	9
X_{10}	Salt concentration (molarity)	0.2	1.8

Table S2. Solvent gradient during HPLC fractionation

Time (min)	Command	Value (%)
0.1	Pump B concentration	0
3.0	Pump B concentration	5
10.0	Pump B concentration	10
20.0	Pump B concentration	50
23.0	Pump B concentration	100
26.0	Pump B concentration	100
28.0	Pump B concentration	0
33.0	Pump B concentration	0
33.0	Stop controller	

Solutions of 0.1% trifluoroacetic acid (TFA) and 70% acetonitrile (ACN) were used as eluent A and B, respectively. Elution was carried out at a flow rate of 1 ml/min.

Table S3. Weighted effects of extraction time sodium chloride concentration and pH on experimental responses expressed in percent

		Julius						*Ponticus*					
Factors	Coeff.	Coeff. Protein	Effect (%)	Coeff. IAA	Effect (%)	Sum of effects (%)		Coeff. Protein	Effect (%)	Coeff. IAA	Effect (%)	Sum of effects (%)	
**Constance**													
	b_0	0.472		71.5				0.406		60.62			
**Linear**													
X_{10}	b_1	-0.071	13.9	-1.0	2.1	8.0	**29.6**	-0.213	14.7	-13.8	22.7	18.7	**44.1**
X_6	b_2	0.086	16.7	-1.7	3.6	10.2		-0.115	8.0	5.1	8.4	8.2	
X_8	b_3	0.102	19.9	-1.4	3.1	11.5		-0.149	10.3	-14.6	24.1	17.2	
**Quadratic**													
$X_{10}*X_{10}$	b_{11}	0.023	4.5	11.4	24.8	14.6	**49.0**	0.089	6.2	-0.2	0.4	3.3	**25.0**
X_6*X_6	b_{22}	-0.047	9.1	11.5	25.1	17.1		0.156	10.8	-14.4	23.7	17.2	
X_8*X_8	b_{33}	0.098	19.1	7.1	15.4	17.2		0.112	7.8	-0.8	1.3	4.5	
**Interaction**													
$X_{10}*X_6$	b_{12}	-0.017	3.4	-1.6	3.5	3.4	**21.4**	-0.208	14.4	-1.9	3.1	8.7	**30.9**
$X_{10}* X_8$	b_{13}	0.031	6.0	-6.7	14.6	10.3		0.049	3.4	-8.8	14.5	9.0	
$X_6* X_8$	b_{23}	-0.039	7.6	3.6	7.8	7.7		0.356	24.6	-1.1	1.9	13.2	
**Total**		0.514	100.0	46.0	100.0	100.0	**100.0**	1.447	100.0	60.8	100.0	100.0	100.0

X_6 is extraction time.X_8 the pH and X_{10} sodium chloride concentrations.

Table S4. Regression coefficients and analysis of variance for protein concentration and IAA from Plackett–Burman design

	Julius				***Poncticus***			
	Protein		*IAA*		Protein		*IAA*	
	Coeff.	*p*-value	Coeff.	*p*-value	Coeff.	*p*-value	Coeff	*p*-value
Constant	0.404		63.79		0.387		59.99	
Type of solvent, X_1	0.010	0.1128	-1.10	0.5492	0.015	0.0659	-0.21	0.7529
Composition of solvent, X_2	-0.003	0.6749	-4.20	0.0376*	0.005	0.4777	-2.31	0.0052*
Ratio samples/solvent, X_3	-0.012	0.0695	4.68	0.0234*	-0.021	0.0172*	1.40	0.0580
Concentration Urea, X_4	0.008	0.2226	3.35	0.0866	-0.001	0.8942	-5.69	0.0000*
Temperature, X_5	0.016	0.0196*	-4.33	0.0332*	-0.008	0.2882	1.07	0.1334
Time, X_6	-0.024	0.0016*	4.72	0.0224*	0.022	0.0128*	-3.76	0.0001*
Stirringspeed, X_7	-0.004	0.4877	3.83	0.0543	-0.021	0.0189*	3.16	0.0006*
pH, X_8	0.015	0.0254*	4.80	0.0206*	0.018	0.0338*	5.35	0.0000*
Type ofsalt, X_9	-0.175	0.0000*	-20.78	0.0000*	-0.137	0.0000*	-	0.0000*
Concentrationofsalt, X_{10}	-0.055	0.0000*	-9.79	0.0089*	-0.039	0.0014*	-5.46	0.0000*
Centrifugationspeed, X_{11}	0.008	0.1788	0.86	0.6392	0.008	0.3112	1.08	0.1308

* Significant values
